# Corneal Biomechanical Properties after FS-LASIK with Residual Bed Thickness Less Than 50% of the Original Corneal Thickness

**DOI:** 10.1155/2018/2752945

**Published:** 2018-01-11

**Authors:** Haixia Zhang, Muhammad Ahmad Khan, Di Zhang, Xiao Qin, Ding Lin, Lin Li

**Affiliations:** ^1^School of Biomedical Engineering, Capital Medical University, Beijing 100069, China; ^2^Beijing Key Laboratory of Fundamental Research on Biomechanics in Clinical Application, Capital Medical University, Beijing 100069, China; ^3^Aier School of Ophthalmology, Central South University, Changsha, Hunan 410015, China; ^4^Changsha Aier Eye Hospital, Changsha, Hunan 410015, China

## Abstract

**Background:**

The changes in corneal biomechanical properties after LASIK remain an unknown but important topic for surgical design and prognostic evaluation. This study aims to observe the postoperative corneal biomechanical properties one month after LASIK with amount of corneal cutting (ACC) greater than 50% of the central corneal thickness (CCT).

**Methods:**

FS-LASIK was performed in 10 left rabbit eyes with ACC being 60% (L60) and 65% (L65) of the CCT, while the right eyes (R) were the control. After 4 weeks, rabbits were executed and corneal strip samples were prepared for uniaxial tensile tests.

**Results:**

At the same strain, the stresses of L65 and L60 were larger than those of R. The elastic moduli of L60 and L65 were larger than those of R when the stress was 0.02 MPa, while they began to be less than those of R when stress exceeds the low-stress region. After 10 s relaxation, the stress of specimens L65, L60, and R increased in turn.

**Conclusion:**

The elastic moduli of the cornea after FS-LASIK with ACC greater than 50% of the CCT do not become less under normal rabbit IOP. The limit stress grows with the rise of ACC when relaxation becomes stable.

## 1. Introduction

The cornea is a significant part of the eye's refractive system, providing approximately 70% of the refractive power. Changes in corneal morphology may alter the refractive state of the eye, causing refractive problems such as myopia. The most common existing surgical approach for the correction of refractive problems is excimer laser corneal refractive surgery [1]. Corneal refractive power is closely related to the geometry of the cornea, which is closely related to corneal biomechanical equilibrium under intraocular pressure (IOP) and atmospheric pressure.

Refractive outcome after corneal refractive surgery is related to the thickness of cornea; early studies have found that the amount of ablation in the corneal stroma is positively correlated with the vision power to be corrected in surgery [2]. Since laser in situ keratomileusis (LASIK) ablates the corneal stroma, in which collagen fibers decrease, the cohesive strength of the cornea must be affected [3]. If the residual collagen fibers were reduced to a certain extent, corneal biomechanical properties may change. It possibly results in corneal forward protrusion [4] and ectasia of the posterior corneal surface [5]. The thinner the thickness of the residual stromal bed is, the easier ectasia could occur [6–8]. Randleman et al. [6] designed the Ectasia Risk Score System, which takes into account the most common risk factors in order of significance, including abnormal preoperative corneal topography, low residual stromal bed thickness, young age, thin preoperative corneal thickness, and higher attempted refractive correction. Current clinical consensus considers the safe residual bed thickness after surgery which should be no less than 250 *μ*m (about 1/2 of the corneal thickness). Too deep or too shallow of the ablation will lead to unsatisfactory vision outcome. In fact, a number of researches have shown that the therapeutic effect of LASIK is influenced by corneal biomechanical properties [9, 10] which might be an important factor for postoperative corneal refractive changes [11, 12].

The researches [13–15] have shown some changes of corneal elastic modulus and creep characteristics after LASIK with different ablation depths. However, the understanding of corneal biomechanical properties after refractive surgery is far from adequate, such as the differences between preoperative and postoperative corneal nonlinear elasticity (viscoelasticity) and their changes along with repair time. In addition, for some special cases, such as higher myopia, whether the clinically secured thickness could be broken through to achieve better visual acuity is the question that people expect to be answered.

The Ocular Response Analyzer (ORA) is one of the clinical devices on the measurement of corneal biomechanics in vivo. The reported studies [16, 17] have suggested CH and CRF obtained by ORA to decrease significantly after refractive surgery. Since there have not been clear biomechanical interpretations of CH and CRF available yet, that is, the relationship between CH, CRF, and corneal biomechanical properties, such as elasticity and viscoelasticity, has not been clear. The comparisons of preoperative and postoperative CH and CRF are not sufficient to understand the changes in corneal biomechanical properties after surgery. Inflation tests [18–20] of the cornea in vitro can maintain its integrity, and the loading mode is close to the physiological conditions of the cornea. Thus, inflation tests are usually considered more reliable than uniaxial tensile tests in studying corneal biomechanics. However, it is not uncommon to study the corneal viscoelastic characteristics after refractive surgery with the inflation test. Moreover, it is complicated to calculate the corneal constitutive parameters from inflation test data, usually by using the finite element method, and it may lead to large cumulative errors. The uniaxial tensile test is the classical mechanical test, which has the advantages of easy implementation, good stability, and simple data processing. The uniaxial tensile test has been used in studies on corneal biomechanical properties [13–15, 18, 19, 21–23].

The purpose of this study is to observe the postoperative corneal nonlinear elasticity and relaxation property one month after FS-LASIK, where the amount of corneal cutting (ACC, the sum of corneal flap thickness and ablation thickness) was designed greater than half of the original corneal thickness, by applying uniaxial tensile tests of corneal strips. And the results will be hoped to enhance our understanding from a biomechanical perspective on some risk factors of LASIK, such as low residual stromal bed thickness and/or high IOP.

## 2. Methods

### 2.1. Specimen Preparation

#### 2.1.1. Experimental Animals

Ten New Zealand white rabbits aged 4 months were selected from the Laboratory Animal Center of Xiangya Medical School of Central South University. The protocol for the experimental animals was approved according to the relevant laws and institutional regulations. All rabbit eyes were examined by slit lamp to exclude anterior segment lesions.

#### 2.1.2. Preoperative Examination and Care

**Figure 1:**
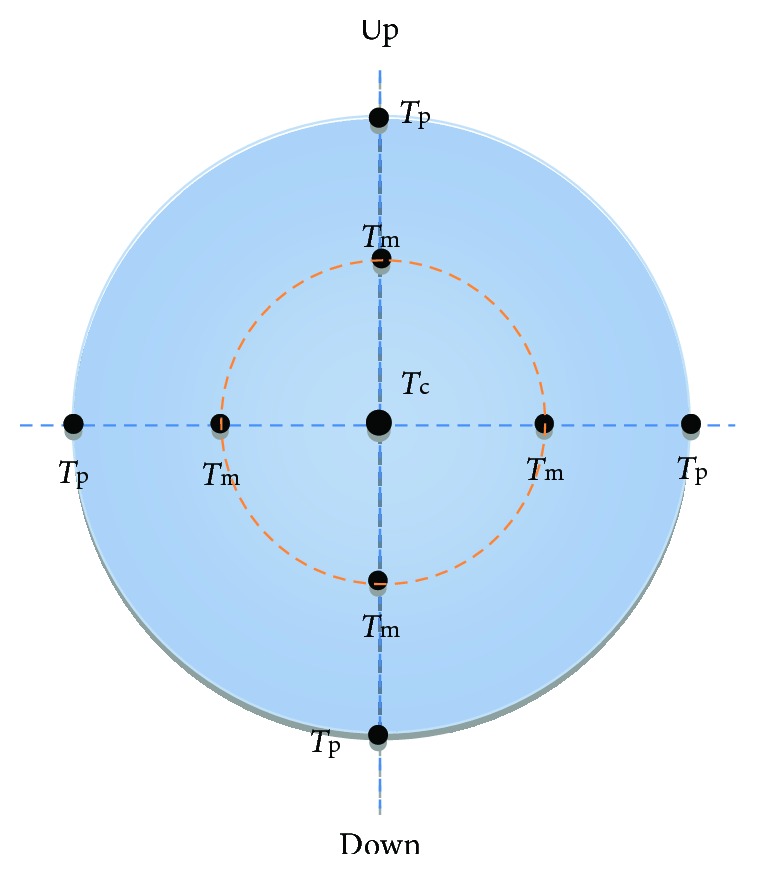
The schematic diagram for the measurement of corneal thickness. *T*_c_ is the measurement point for CCT, *T*_m_ is the measurement point for MCT, and *T*_p_ is the measurement point for PCT.

After topical anesthesia with oxybuprocaine 20 mg/80 mg solution (Alcon, United States), we used Pachymeter SP3000 (TOMEY, Japan) to measure the central corneal thickness (CCT), the midperipheral corneal thickness (MCT), and the peripheral corneal thickness (PCT). As shown in Figure 1, the measurement point of CCT (*T*_c_) is at the apex of the cornea. The measurement points of PCT (*T*_p_) are at the outer margin of the cornea, that is, the limbus. The measurement points of MCT (*T*_m_) are the midpoints of the corneal apex and the limbus, and the distance between *T*_c_ and *T*_m_ is about 3-4 mm. Each point was measured three times, and the average value was used. All rabbits were applied slit lamp observation to exclude eye disease, and 0.3% tobramycin dexamethasone eye drops (Alcon, United States) were applied to all rabbits 2 days before surgery to prevent infection.

#### 2.1.3. Surgical Procedures

**Figure 2:**
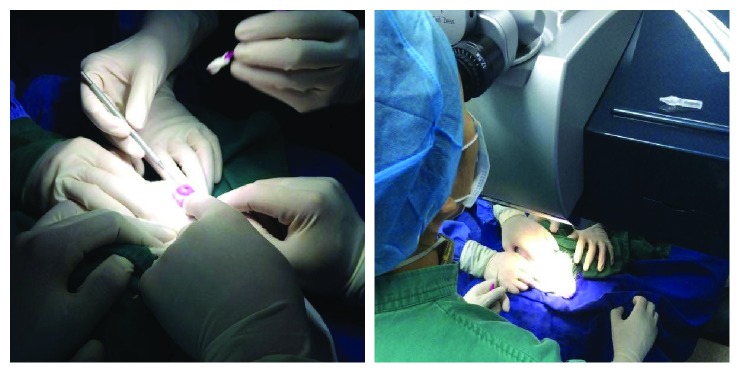
Duration of opening of the corneal flap (left) and excimer laser ablation (right).

General anesthesia of the rabbits was performed with ketamine 5 mg/kg intramuscular injection, and all rabbits were then placed on the operating table for conjunctival sac washing with 5% of iodine. FS-LASIK surgery was performed in the left eyes of the rabbits by professional ophthalmologists of Aier Eye Hospital (Figure 2), and the right eyes were taken as the blank control. Corneal flap thickness was set at 120 *μ*m (all flap pedicles were located in the upper portion of the eyes), excimer laser cutting energy at the corneal stromal bed at 1.0 *μ*J, femtosecond laser scanning line distance at 8 *μ*m, spot pitch at 8 *μ*m, and flap edge cutting energy at 1.3 *μ*J. After flap formation, corneal stromal bed thickness was examined with the flap that was overturned. An excimer laser of 193 nm wavelength (SCHWIND AMARIS laser, Germany) was aligned and positioned to the corneal center, and cutting was performed. For the SCHWIND AMARIS laser, 1-diopter correction roughly needs the ablation of 12 *μ*m corneal thickness. We converted the needed thickness of ablation into the equal diopter correspondingly. We took the calculated diopter as initial input values, and the machine gave a residual bed thickness. We changed the value of the diopter until we obtained the designed residual bed thickness. BSS liquid (Shenyang Pharmaceutical Co. Ltd. Qi) was used afterwards to flush the cutting surface, and the corneal flap was repositioned and paired. 0.3% tobramycin dexamethasone eye drops were then applied, and transparent eyepatches were put on, thus after the surgery was finished. The residual corneal thickness of rabbit numbers 1–5 was 35% of the original corneal thickness (i.e., ACC took up 65% of the original thickness; Table 1) (L65), and the residual corneal thickness of rabbit numbers 6–10 was 40% (L60). All the right eyes without FS-LASIK surgery were taken as the control group (R).

#### 2.1.4. Postoperative Care and Testing

All rabbits were raised and taken care of at the Laboratory Animal Center of Xiangya Medical School, Central South University. 0.3% tobramycin dexamethasone eye drops 4 times a day were applied to all surgical eyes within the first week after surgery and were reduced to twice a day during postoperative 1 week to 1 month.

#### 2.1.5. Preparation of Corneal Strips

Rabbits were anesthetized to death through an intravenous injection of a 25% urethane agent via the rabbit auricular vein at the 4th week. In order to make the corneal strips at the same position and the same orientation, we marked the eyeballs at 6 and 12 o'clock direction after the rabbits died. The eyeballs were then taken out, and contents of lens, iris, and vitreous body were removed within half an hour. Corneal strips were prepared with a double-edged knife along the 6 and 12 o'clock direction (the up–down direction). Strip length and width were measured with a vernier caliper. The morphologies of postoperative corneas were observed via Visante OCT (Zeiss, Germany) at the second weekend and before the execution of the animals. Significant corneal ectasia was not found.

### 2.2. Uniaxial Tests

**Figure 3:**
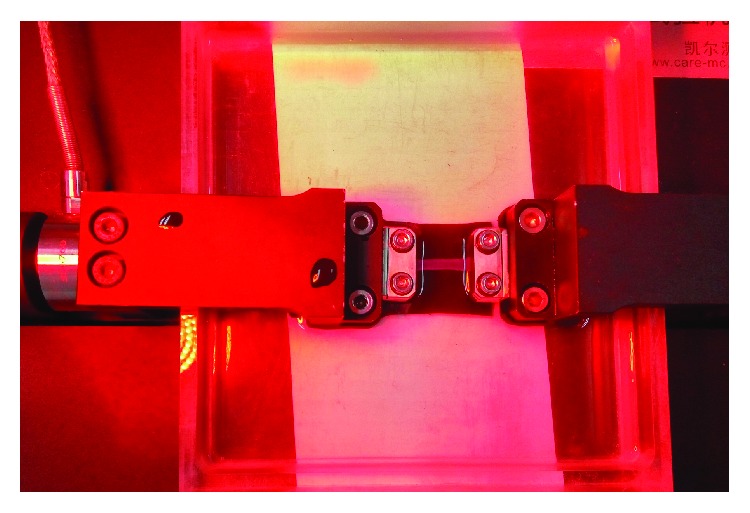
Clamping and water bath system of corneal strip.

**Figure 4:**
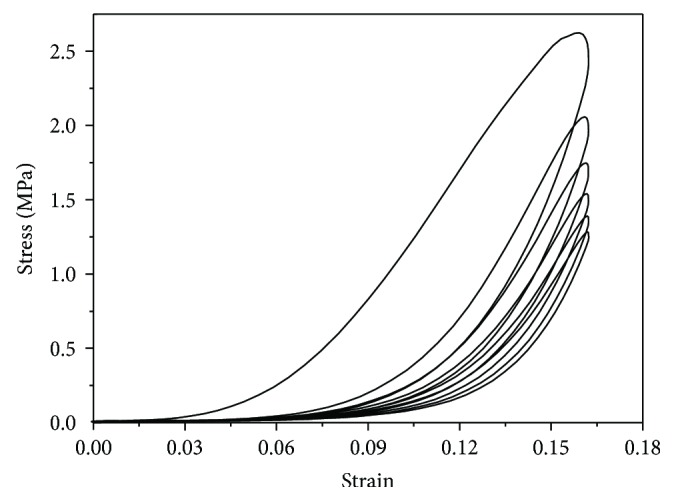
Stress-strain data during the preconditioning process. The hysteresis loop decreases with succeeding cycles.

We used the Care-IBTC-50 (in situ bidirectional tension and compression) Testing System (CARE Measurement & Control Corp., Tianjin, China) to perform uniaxial tensile tests (Figure 3) at room temperature. Corneal strips were kept moist with saline bath. Each of the specimens was subjected to a set of loading and unloading uniaxial tension; it was seen that the hysteresis loop decreased between successive cycles (Figure 4). After 6 cycles, the stress-strain curve became stable and the specimens were regarded as preconditioned; the stress-strain test at a tensile rate of 0.02 mm/s was carried out afterwards. After a 5-minute recovery, all corneal strips were stretched at a rate of 0.5 mm/s till they became 125% of the original length and a 10-minute stress-relaxation test was performed.

### 2.3. Stress-Strain Relationship and Relaxation Function

The exponential model can well describe the relationship between stress and strain in uniaxial tension of soft tissue [22–27]. Assume that the stress-strain relationship of the corneal strip is governed by the exponential model.

(1) $$\begin{array}{c} \sigma =A\left(e^{B\varepsilon }-1\right). \end{array}$$

Before fitting the experimental data, we need to prepare the formula due to thickness in homogeneity of the corneal strips in the L65 and L60 groups. Let the original length of the strip be *L* = *L*_*r*_ + 2*L*_c_, where *L*_*r*_ is the diameter of the cutting area and *L*_c_ is the length of the corneal strip from the cutting edge to the corneoscleral limbus. Therefore, the length of elongation is Δ*L* = Δ*L*_*r*_ + 2Δ*L*_c_.

**Figure 5:**
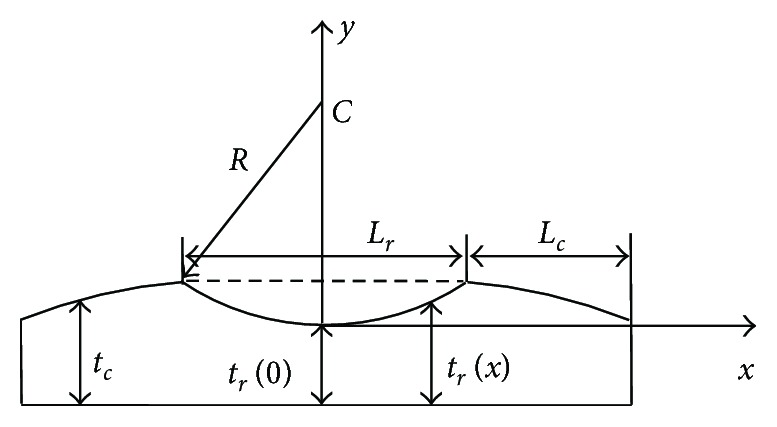
Schematic diagram of the corneal section.

By (1), the following equations can be obtained:

(2) $$\begin{array}{c} \sigma_{r}=A\left(e^{B\varepsilon_{r}}-1\right),\\ \sigma_{c}=A\left(e^{B\varepsilon_{c}}-1\right), \end{array}$$

where *σ*_*r*_ = *F*/(*t*_*r*_*w*_0_), *σ*_c_ = *F*/(*t*_c_*w*_0_), and *w*_0_ is the width of the corneal strip and *t*_*r*_ and *t*_c_ are the thicknesses of the cutting area and other area of the corneal strip. It should be considered that *t*_c_ almost is constant, where we took it as PCT, and that *t*_*r*_ is variable, which is related to location *x* (Figure 5). Therefore,

(3) $$\begin{array}{c} \varepsilon_{r}=\frac{1}{B}\log\left[1+\frac{\sigma_{r}\left(x\right)}{A}\right]=\frac{1}{B}\log\left[1+\frac{F}{{Aw}_{0}t_{r}\left(x\right)}\right], \end{array}$$

and the length of elongation for the cutting area gives

(4) $$\begin{array}{c} \Delta L_{r}=\int_{-\left(L_{r}/2\right)}^{L_{r}/2}\varepsilon_{r}\left(x\right)dx=\int_{-L_{r}/2}^{L_{r}/2}\frac{1}{B}\log\left[1+\frac{F}{{Aw}_{0}t_{r}\left(x\right)}\right]dx, \end{array}$$

where $t_{r}\left(x\right)=t_{r}\left(0\right)+R-\sqrt{R^{2}-x^{2}},R=\left({L_{r}}^{2}/4+\delta^{2}\right)/\left(2\delta \right),\text{and }\delta =CCT-t_{r}\left(0\right)$. Following the method in [15], we fit the experimental data of the L60 and L65 groups. For the data of the R group, (1) can directly be used where the stress *σ* = *F*/(*t*_c_*w*_0_) and the thickness *t*_c_ can be taken as the CCT of the cornea, neglecting the difference between the central and the midperipheral corneal thicknesses.

**Figure 6:**
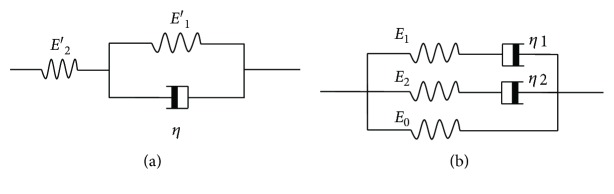
Schematic representation of the three-element model (a) and the Maxwell-Wiechert model (b).

The corneal viscoelastic properties could be described by a linear viscoelastic model [24]. In this study, we used the three-element model which combines the Voigt model with a Hookean spring in series (Figure 6(a)) and the generalized Maxwell model also known as the Maxwell-Wiechert model that combines two Maxwell elements and a Hookean spring in parallel (Figure 6(b)).

For the three-element model and the Maxwell-Wiechert model, the governing constitutive relations were

(5) $$\begin{array}{c} \frac{d\sigma \left(t\right)}{dt}+\frac{E_{1}^{\prime }+E_{2}^{\prime }}{\eta }\sigma \left(t\right)=E_{2}^{\prime }\frac{d\varepsilon \left(t\right)}{dt}+\frac{E_{1}^{\prime }E_{2}^{\prime }}{\eta }\varepsilon \left(t\right), \end{array}$$

and

(6) $$\begin{array}{c} \sigma \left(t\right)=\sigma_{0}\left(t\right)+\sigma_{1}\left(t\right)+\sigma_{2}\left(t\right),\\ \frac{1}{E_{1}}\frac{d\sigma_{1}\left(t\right)}{dt}+\frac{1}{\eta_{1}}\sigma_{1}\left(t\right)=\frac{d\varepsilon \left(t\right)}{dt},\\ \frac{1}{E_{2}}\frac{d\sigma_{2}\left(t\right)}{dt}+\frac{1}{\eta_{2}}\sigma_{2}\left(t\right)=\frac{d\varepsilon \left(t\right)}{dt},\\ \sigma_{0}\left(t\right)=E_{0}\varepsilon \left(t\right), \end{array}$$

where *σ*(*t*) and *ε*(*t*) were the stress and strain, respectively, at time *t*, *E*_1_′ and *E*_2_′ were the elastic moduli of the springs, and *η* was the viscosity of the dashpot in Figure 6(a). *σ*_0_, *σ*_1_, and *σ*_2_ were the stresses of the spring and two Maxwell elements (Figure 6(b)), respectively. *E*_0_, *E*_1_, and *E*_2_ were the elastic moduli of the springs, and *η*_1_ and *η*_2_ were the viscosities of the two dashpots in Figure 6(b).

In the stress-relaxation state *dε*/*dt* = 0, according to the solutions of differential equations (5) and (6), the following result could be obtained separately:

(7) $$\begin{array}{c} G\left(t\right)=\frac{E_{e}}{E_{2}^{\prime }}+\frac{E_{e}}{E_{1}^{\prime }}e^{-\left(t/t_{s}\right)},\;E_{e}=\frac{E_{1}^{\prime }E_{2}^{\prime }}{E_{1}^{\prime }+E_{2}^{\prime }}, \end{array}$$

(8) $$\begin{array}{c} G\left(t\right)=\frac{E_{0}}{E_{s}}+\frac{E_{1}}{E_{s}}e^{-\left(t/t_{1}\right)}+\frac{E_{2}}{E_{s}}e^{-\left(t/t_{2}\right)},\;E_{s}=E_{0}+E_{1}+E_{2}, \end{array}$$

where *G*(*t*) = *σ*(*t*)/*σ*(0) is the normalized stress-relaxation function. Let *t* → ∞ the limit of *G*(*t*) give the stable value of stress-relaxation, that is, *G*_∞_ = *E*_*e*_/*E*_2_′ and *G*_∞_ = *E*_0_/*E*_*s*_ from (6) and (7), respectively. *t*_1_ = *η*_1_/*E*_1_, *t*_2_ = *η*_2_/*E*_2_, and *t*_*s*_ = *η*/(*E*_1_′ + *E*_2_′) were relaxation time constants. Models (7) and (8) are also known as 1- and 2-item Prony series models.

## 3. Results

### 3.1. In Vivo Test Data

The preoperative corneal thickness of the rabbit eye was measured by a pachymeter and OCT, while the postoperative corneal thickness was measured only by OCT. In Table 1, preoperative corneal thickness (CCT, MCT, and PCT) and postoperative CCT were shown. According to our previous research [28], there was no significant difference between CCT measured by a pachymeter and OCT images, and in this study, we also got no significant difference in results shown in Table 1. Moreover, CCT measured from OCT images was more accurate in post-LASIK eyes than that measured from other measurements [29]. Thus, for postoperative CCT, we used the values measured from OCT images. In this study, the sum of residual bed thickness and corneal flap thickness was postoperative CCT, which has a significant difference between the L65 and L60 groups (see Table 1) (*p* = 0.030 < 0.05). Thus, we used the designed values of the cutting thickness to group experimental animals and to process the uniaxial tensile test data.

One-way ANOVA showed that there were no significant differences among CCT, MCT, and PCT (*p* = 0.430 > 0.05). Therefore, it was reasonable that the thickness of the R group in (1) can be taken as the CCT of the cornea, neglecting the difference between the central and the midperipheral corneal thicknesses.

### 3.2. Stress-Strain Experimental Data

The length and width of corneal strips for the L65 group, L60 group, and R group are listed in Table 2. One-way ANOVA showed that there were no significant differences among the L65 group, L60 group, and R group for length (*p* = 0.915 > 0.05), width (*p* = 0.319 > 0.05), and length-width ratio (*p* = 0.503 > 0.05).

**Figure 7:**
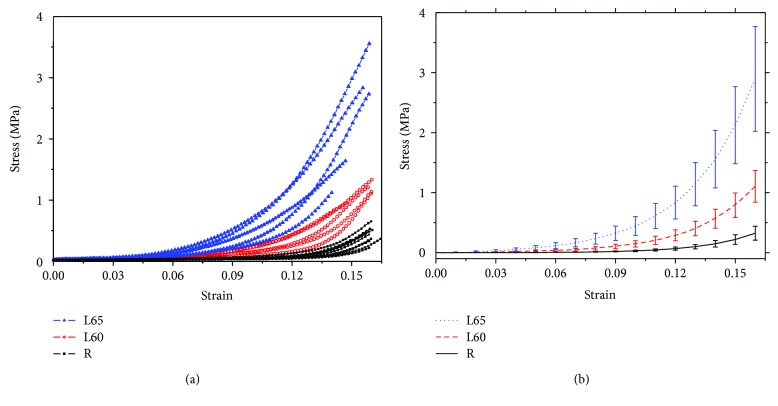
Stress-strain data (a) and its theoretical fit (b) of corneal strips.

**Figure 8:**
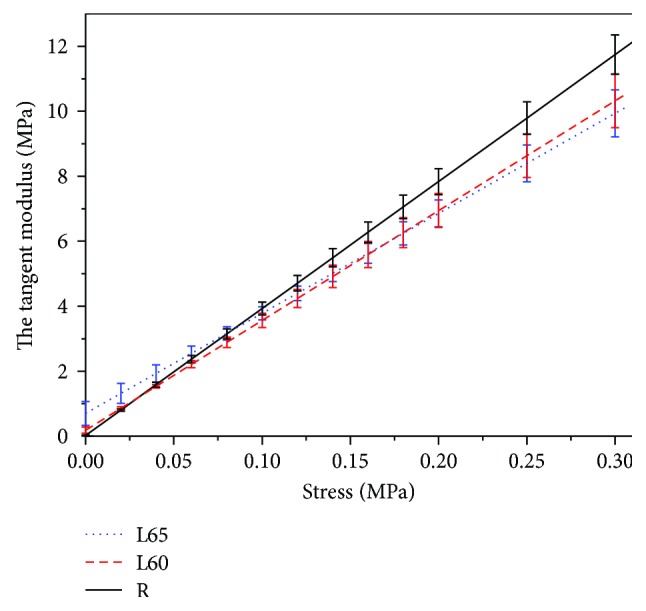
The stress-tangent modulus curves of corneal strips.

Figure 7(a) shows the stress-strain data of the R group (black squares), L60 group (hollow circles), and L65 group (blue triangles). The fitting results (Table 3) to the data were expressed as mean ± standard deviations. The goodness-of-fit *R*^2^ was greater than 0.98.  Figure 7(b) shows the fitting results of the L60, L65, and R groups.  Figure 8 shows the corresponding stress-tangent modulus curves.

It can be seen from Figure 7(a) that the stress-strain curve of each group appears to be nonlinear and has a typical “J curve” feature. At the same strain level, the stress of L65 (L60) is greater than that of the R group. We further investigated the differences between the parameters *A* and *B* in model (1) of the corresponding sample groups. The paired *t*-test gave that both *A* and *B* between the left cornea (surgery) group and the right cornea (control) group showed significant difference (*p* = 0.0102, 0.0005 < 0.05). Independent sample *t*-test showed that there were significant differences between the L60 group and the L65 group for parameter *A* (*p* = 0.026 < 0.05) and there were no significant differences between the L60 group and the L65 group for parameter *B* (*p* = 0.174 > 0.05). The difference of the corresponding parameters among groups also indicated the significant difference in the stress-strain relationship between different groups.

Stress-tangent modulus curves in Figure 8 show that tangent modulus increases with the rise of stress, wherein the tangent modulus of R grows faster with the increase in stress than those of L60 and L65. At the same stress level within the range of low stress (stress < 0.03 MPa, equivalent to 20.1 mmHg normal rabbit IOP, calculated from the formula *σ* = *P* × *r*/(2*t*_c_), where the radius *r* of the adult rabbit cornea is approximately 7.5 mm and the thickness *t*_c_ of the cornea is about 0.33 mm), tangent modulus of L65 is greater than that of L60, which is greater than that of R slightly. When stress exceeds this range, tangent modulus in each group exhibits larger variations with the rise of stress. The tangent moduli of L65 and L60 begin to be less than that of the R group. In a high-stress region (stress > 0.08 MPa, equivalent to 53.5 mmHg normal rabbit IOP), tangent moduli of the different groups present an opposite distribution to those in the range of low stress; that is, at the same stress level, tangent modulus of R is greater than that of L60, which is greater than that of L65. If we considered tangent modulus in the physiological level of IOP 15–30 mmHg, that is, in the range of 0.025–0.05 N load [15], our results gave 1.0590 ± 0.1657 MPa for the L65 group, 0.7668 ± 0.0958 MPa for the L60 group, and 0.3582 ± 0.0519 MPa for the R group.

### 3.3. Stress-Relaxation Data

**Figure 9:**
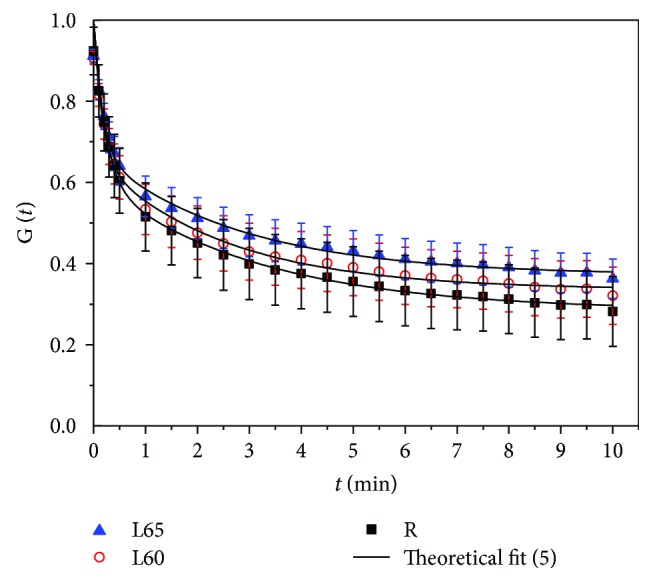
Stress-relaxation curves of corneal strips.

Figure 9 shows the averaged data of normalized stress-relaxation for each group. The stress-relaxation data indicated that the stress-relaxation rate is rapid in the 1st minute after stress-relaxation started and then becomes slower. The stress-relaxation at the same time point is in descending order of R, L60, and L65 at reducing amplitude of 1.1 times. The stresses of specimens L65, L60, and R were 29.60%, 33.58%, and 37.60%, respectively, of the initial stress after 10 s relaxation. The times required for stress-relaxation to half of the initial stress of the specimens R, L60, and L65 were 1.23 min, 1.61 min, and 2.36 min, respectively.

In the form of mean ± standard deviation, Table 4 gives the fitting results of stress-relaxation data for each group obtained by three-parameter model (7). And the goodness-of-fit *R*^2^ is all greater than 0.78.  Table 5 displays the fitting result of stress-relaxation values from (8) with the goodness-of-fit *R*^2^ greater than 0.97. The fitted model parameters *E*_0_, *E*_1_, *E*_2_, *t*_1_, and *t*_2_ were analyzed statistically. Through the rank sum test, differences of parameters *E*_0_, *E*_1_, and *E*_2_ among L60, L65, and R are significant (*p* < 0.05), while differences of *t*_1_ and *t*_2_ are not (*p* > 0.05). We also note that the stress (normalized) value *G*_∞_ (Tables 4 and 5) of the corneal strip also grows with the rise of ACC when relaxation becomes stable.

## 4. Discussion

Researchers generally believe that corneal biomechanical properties may be an important factor to postoperative corneal refractive changes. In this study, FS-LASIK was performed in rabbits with ACC at 60% and 65% of the original corneal thickness and all rabbits were raised postoperatively for 4 weeks. Thereafter, the corneal strips were made from the executed rabbits, and the mechanical test was performed. The experimental data of stress-strain and stress-relaxation under different ACC was analyzed to study the influence of surgical ACC on corneal biomechanical properties.

The cornea as a soft tissue exhibits a nonlinear stress-strain characteristic. Our experimental data shows that the stress-strain curve of the corneal strip in each group exhibits a typical “J” shape. It means that the elastic moduli of the cornea are different for different stress regions. Since we designed to perform the relaxation test after the tensile test, the elastic limit of the cornea was not obtained. However, we have noted that the uniaxial tensile stress, experienced by the cornea whose ACC is less than 65% of the original, namely, more than 35% remains of the residual strip thickness, can be greater than 2.5 MPa. At the same level of strain, the stress in L65 is more than twice the stress in L60, while the stress in L60 is double that of group R. The larger the amount of ablation, the greater the corneal stress at the same strain. The possible reasons are the following. The postoperative cornea would undergo a process for establishing a new morphological stability due to the IOP. The large amount of ablation results in a thin central cornea. Therefore, when the new morphological stability is achieved, the stress will be greater in the cornea experiencing larger ACC than in the cornea with smaller ablation.

In this study, (1), one of the commonly used one-dimensional constitutive models, was used to describe the tensile result of each strip group. Table 3 shows the better fits to the axial tension data of corneal strips from the rabbit cornea after refractive surgery. We note that the fitting results of model (1) are quite consistent with *A* = 0.00019 MPa and *B* = 33.65 [27] and *A* = 0.0009 MPa and *B* = 23.74 [22]. Table 3 also suggests that statistically significant differences exist among L65, L60, and R groups for parameters *A* and *B* and that with the increase in ACC, parameter *A* increases and parameter *B* decreases. Nash et al. [25] report that keratoconus has a low *B* and a high *A* value with respect to the normal cornea. Combining our results with the results of [25], we inferred that the greater the amount of ablation, the higher the risk of corneal keratoconus. But the deduction should be investigated through long-period follow-up. Stress-strain data shown in Figure 7 also displays the difference of stress-strain features among groups. Therefore, there are true differences of corneal stress-strain relationship 4 weeks after surgery among the FS-LASIK of different ACC.

According to [15], where the elastic modulus is in the range of 0.025–0.05 N load (approximately as the physiological level of IOP 15–30 mmHg), there was a significant increase in the elastic modulus of corneal strips in the operation group. The present results show that the elastic moduli in the range of 0.025–0.05 N load of the corneal strips in L65 (1.0589 ± 0.1657 MPa) and L60 (0.7668 ± 0.0958 MPa) are about 2.9 and 2.1 times, respectively, that in the R group. Both two studies showed that the elastic moduli of corneal strips in the operation group are larger than that in the normal group. In addition, based on the in vitro corneal inflation test after photochromic keratectomy of the rabbit cornea, [4] reports the rise of elastic modulus with the increase in cutting depth under low pressure (less than 30 cm H_2_O, about 21.9 mmHg). Our results show that tangent moduli of corneal strips in L65 are greater than those in L60, which are greater than those in R slightly within the low-stress region. From these statements, we see some consistent results from those studies, and the possible reasons for the increase in elastic modulus with deeper ablation after 4 weeks postoperatively have been discussed in [15] based on Davis's law, which is used to describe how soft tissue models along imposed demands. It is a physiological principle stating that soft tissue heals according to the manner in which they are mechanically stressed [30]. Furthermore, we noticed that there are a few of new information. The present study gives that when stress exceeds the low-stress region (20.1 mmHg normal rabbit IOP, approximately), the tangent moduli of L65 and L60 begin to be less than those of the R group. It means that low residual stromal bed thickness from excessive ablation is a risky factor when IOP become higher. The possible reason is more fibrils in the normal cornea than the residual stromal bed of the ablated cornea.

Stress-relaxation data shows that the rate of stress-relaxation is rapid within 1 minute of the stress-relaxation period and slows down afterwards. The stable value of stress-relaxation of corneal strips decreases with the increase in ACC, which is consistent with the result [31]. But differences in stress-relaxation among different ACC in [31] are greater than those in our experiment. The reason might lie in the different fibrous structures in the pig and rabbit cornea or may be caused by the in vitro corneal ablation of experimental samples in literature [31] while our experiment specimens come from living rabbits 4 weeks after FS-LASIK. In addition, according to the concept of stress-relaxation and the fact that *G*_∞_ of L65, L60, and R decreased in subsequent turn (Figure 9), we knew that, during a long time, the transient stresses in the strips of the ablated corneas were larger than those in corneal strips without surgery. On the other hand, the formula in [32], *σ*_c_ + 2*σ*_*t*_*rt*/*a*^2^ = (*r*/*a*)^2^*p*, gives the relationship of real IOP and its measurement value. In the formula, *σ*_c_ is the compressive contact stress, that is, the IOP measurement value, and *a*, *r*, *t*, *p*, and *σ*_*t*_ are the radius of the flattened area on the cornea by *σ*_c_, corneal radius, corneal thickness, real IOP, and tensile stress, respectively. From the clinical data, we know that the IOP measurement values by Goldmann tonometry and IOPg obtained from the Ocular Response Analyzer (ORA) decreased significantly after refractive surgery [33–35]. If we assume that the real IOP of a rabbit's eye are stable and the corneal deformation after refractive surgery was actually not large except CCT, it can be inferred that the corneal stress *σ*_*t*_ in vivo after refractive surgery is greater than that of the nonsurgical cornea. Our results are consistent with the above results.

In this study, two linear viscoelastic models, three-element model, and Maxwell-Wiechert model were used to study stress-relaxation of the cornea. The results show a higher goodness of fit in the Maxwell-Wiechert model (*R*^2^ > 0.99) than that in the three-element model. Su et al. [36] used a 4-item Prony series model, namely, the Maxwell-Wiechert model with 4 sets of the Maxwell model, to fit the stress-relaxation data of porcine corneal strips and obtained a high goodness of fit. However, considering the number of model parameters, we think that the Maxwell-Wiechert model with two Maxwell elements, that is, the 2-item Prony series model, can better describe the data of postoperative corneal stress-relaxation.

Keeping the decreases [16, 17] in CH and CRF postoperatively in mind, from the results of the present study, that is, the elastic moduli of the operated specimen were larger than those of the controlled specimen (see also [13–15]) and stress-relaxation of the operation groups was slower than that of the control group; we may speculate that smaller CRF and CH may correspond to larger elastic modulus and slower stress-relaxation. Of course, this conjecture requires further experimental verification.

The results of the present study give that in a normal IOP range, the thinner the residual bed thickness is, the greater the corneal elastic modulus is and the slower the stress-relaxation becomes. It means that the elastic moduli of corneal specimens after FS-LASIK with ACC greater than 50% of the original corneal thickness do not become less, and their viscoelastic properties are close to those of the control group. However, the elastic moduli of postoperative corneas decrease in the higher IOP range. It might be inferred that the risk of corneal ectasia will increase if the IOP exceed the normal values after the corneal refractive surgery. Following this line, it is necessary to control the IOP after the refractive surgery and during the self-repair process.

The limitations of this study include the following: (1) The corneal strips of this study were derived from rabbit corneas in vitro after 4 weeks of refractive surgery. The data to evaluate in vivo corneal biomechanical properties cannot be used directly. As we have known, the biomechanical interpretation of ORA output parameters has not been clear yet. It is difficult to understand in vivo corneal biomechanical properties after refractive surgery. The results of this paper show the difference of biomechanical properties between the L65, L60, and R groups of rabbits 4 weeks after FS-LASIK, and experimental conditions of each group are completely the same; thus, the results of this study by uniaxial tests of corneal strips have significances for understanding the biomechanical properties of the cornea postoperatively. (2) The anisotropic biomechanical behavior of the cornea is not observed in this paper. (3) This article reveals the difference of biomechanical properties in rabbit corneal strips 4 weeks after in vivo refractive surgery with different ACC, and no further analysis is made for the underlying causes of these differences. Since biomechanical properties of the cornea are related to the distribution of collagen fibers, a study of collagen fiber distribution can therefore help better understand the changes of corneal biomechanical properties [37]. Studies on corneal fibrous structure are necessary to further analyze the underlying causes for biomechanical changes after LASIK at different ACC. (4) This study deals only with the biomechanical properties after FS-LASIK with 2 close ACC greater than half of the CCT, compared with the three groups with different ablation depths [15]. During 4 weeks after refractive surgery, there had not been observed rabbit corneal ectasia. Therefore, for further investigation of the susceptibility of keratoconus formation when ACC is greater than 1/2 of the original thickness, studies on corneal biomechanical properties with deeper ablation depths and longer observation time are needed. Although this study together with the work of [15] has positive significances for the exploration of the best ACC in clinical practice, there are still a lot of studies that need to be done further. The possible effective approach is to determine the optimal critical value of LASIK ablation through finite element modelling, in combination with the study on the collagen fiber state.

Though this study has limitations, we may conclude that there were changes in the biomechanical properties of the cornea after refractive surgery, and the changes were correlated with the residual bed thickness. The elastic moduli of corneal strips after FS-LASIK with ACC greater than 50% of the original corneal thickness do not become less under normal rabbit IOP. The viscoelastic properties of the surgery group are close to those of the control group, and the limit stress grows with the rise of ACC when relaxation becomes stable. Taking into account the midterm or long-term outcomes of corneal refractive surgery, we have not made sure whether high IOP is a risk factor for corneal ectasia, and further studies, such as the observations on corneas after LASIK with increased IOP, and clinical observations should be designed and carried out.

## Figures and Tables

**Table 1:** Corneal thickness for each group.

Group	CCT (*μ*m)			MCT (*μ*m)	PCT (*μ*m)
	Preoperative Measured by a pachymeter	Preoperative Measured by OCT	Postoperative Measured by OCT	Preoperative Measured by a pachymeter	Preoperative Measured by a pachymeter
L65	335 ± 21	334.5 ± 23.0	227.9 ± 60.6	322 ± 18	327 ± 27
L60	346 ± 10	344.1 ± 6.2	283.8 ± 44.0	335 ± 14	346 ± 10
R	330 ± 15	334.2 ± 13.5	—	329 ± 16	329 ± 16

**Table 2:** The length and width of corneal stripes for each group.

Group	Length (mm)	Width (mm)	Length-width ratio
L65	14.40 ± 0.38	2.97 ± 0.19	4.87 ± 0.35
L60	14.63 ± 1.02	3.00 ± 0.19	4.89 ± 0.54
R	14.53 ± 0.93	3.21 ± 0.40	4.59 ± 0.61

**Table 3:** The fitted parameters of stress-strain data.

Group	*A* (MPa)	*B*	*R* ^2^
L65	0.0239 ± 0.0146	30.7863 ± 3.3343	0.9968 ± 0.0053
L60	0.0057 ± 0.0032	33.7948 ± 3.0346	0.9976 ± 0.0029
R	0.0007 ± 0.0004	39.0638 ± 2.0423	0.9934 ± 0.0033

**Table 4:** The fitted parameters of stress-relaxation data by model (7).

Group	*E*_1_′ (MPa)	*E*_2_′ (MPa)	*t_s_* (min)	*G*_∞_	*R* ^2^
L65	10.1408 ± 5.8173	16.4475 ± 5.8594	0.6501 ± 0.1888	0.4174 ± 0.0479	0.7877 ± 0.0257
L60	5.9513 ± 3.0988	9.1808 ± 2.9271	0.7275 ± 0.0806	0.3785 ± 0.0676	0.8254 ± 0.0134
R	1.2904 ± 0.6890	2.6453 ± 1.0379	0.8094 ± 0.2709	0.3273 ± 0.0706	0.8495 ± 0.0416

**Table 5:** The fitted parameters of stress-relaxation data by model (8).

Group	*E*_1_ (MPa)	*E*_2_ (MPa)	*E*_0_ (MPa)	*t*_1_ (min)	*t*_2_ (min)	*G*_∞_	*R* ^2^
L65	4.4116 ± 1.5107	5.1094 ± 2.0502	5.0964 ± 2.0715	1.9127 ± 1.5894	1.3507 ± 1.6128	0.3478 ± 0.0542	0.9962 ± 0.0006
L60	2.7766 ± 0.9990	2.5855 ± 0.7133	2.7971 ± 1.1720	1.3286 ± 1.5633	1.9579 ± 1.6412	0.3368 ± 0.0631	0.9962 ± 0.0007
R	1.0193 ± 0.5170	0.9638 ± 0.4222	0.8203 ± 0.6567	1.0008 ± 1.1996	2.3151 ± 1.5689	0.2738 ± 0.0685	0.9960 ± 0.0007

## Abbreviations

FS-LASIK: Femtosecond laser in situ keratomileusis
ACC: Amount of corneal cutting
IOP: Intraocular pressure
CCT: Central corneal thickness
MCT: Midperipheral corneal thickness
PCT: Peripheral corneal thickness
ORA: Ocular Response Analyzer
CH: Corneal hysteresis obtained by ORA
CRF: Corneal resistance factor given by ORA.
